# Genome-Wide Identification of Key Components of RNA Silencing in Two *Phaseolus vulgaris* Genotypes of Contrasting Origin and Their Expression Analyses in Response to Fungal Infection

**DOI:** 10.3390/genes13010064

**Published:** 2021-12-27

**Authors:** Juan C. Alvarez-Diaz, Manon M. S. Richard, Vincent Thareau, Gianluca Teano, Christine Paysant-Le-Roux, Guillem Rigaill, Stéphanie Pflieger, Ariane Gratias, Valérie Geffroy

**Affiliations:** 1 Université Paris-Saclay, CNRS, INRAE, Université Evry, Institute of Plant Sciences Paris-Saclay (IPS2), 91405 Orsay, France; juan-camilo.alvarez-diaz@universite-paris-saclay.fr (J.C.A.-D.); manon.ms.richard@gmail.com (M.M.S.R.); vincent@reseau-aae.org (V.T.); gianluca.teano1@universite-paris-saclay.fr (G.T.); christine.paysant-le-roux@inrae.fr (C.P.-L.-R.); guillem.rigaill@inrae.fr (G.R.); stephanie.pflieger@universite-paris-saclay.fr (S.P.); ariane.gratias-weill@universite-paris-saclay.fr (A.G.); 2 Université de Paris, Institute of Plant Sciences Paris Saclay (IPS2), 91405 Orsay, France; 3 Molecular Plant Pathology, Swammerdam Institute for Life Sciences (SILS), University of Amsterdam, 1098 XH Amsterdam, The Netherlands; 4 Laboratoire de Mathématiques et Modélisation d’Evry, Université Paris-Saclay, CNRS, Université Evry, INRAE, 91037 Evry, France

**Keywords:** *Phaseolus vulgaris*, *Colletotrichum lindemuthianum*, RNA silencing, Argonaute, double-stranded RNA binding (DRB), RNA-dependent RNA polymerase (RDR), Pol IV

## Abstract

RNA silencing serves key roles in a multitude of cellular processes, including development, stress responses, metabolism, and maintenance of genome integrity. Dicer, Argonaute (AGO), double-stranded RNA binding (DRB) proteins, RNA-dependent RNA polymerase (RDR), and DNA-dependent RNA polymerases known as Pol IV and Pol V form core components to trigger RNA silencing. Common bean (*Phaseolus vulgaris*) is an important staple crop worldwide. In this study, we aimed to unravel the components of the RNA-guided silencing pathway in this non-model plant, taking advantage of the availability of two genome assemblies of Andean and Meso-American origin. We identified six *Pv*DCLs, thirteen *Pv*AGOs, 10 *Pv*DRBs, 5 *Pv*RDRs, in both genotypes, suggesting no recent gene amplification or deletion after the gene pool separation. In addition, we identified one *Pv*NRPD1 and one *Pv*NRPE1 encoding the largest subunits of Pol IV and Pol V, respectively. These genes were categorized into subgroups based on phylogenetic analyses. Comprehensive analyses of gene structure, genomic localization, and similarity among these genes were performed. Their expression patterns were investigated by means of expression models in different organs using online data and quantitative RT-PCR after pathogen infection. Several of the candidate genes were up-regulated after infection with the fungus *Colletotrichum lindemuthianum*.

## 1. Introduction

Small RNAs have regulatory roles in a multitude of biological processes, including stress responses, development, metabolism, and maintenance of genome integrity, in a sequence-specific manner [1]. Although heterogeneous in size, sequence, genomic distribution, biogenesis, and action, most of these small RNA molecules mediate repressive gene regulation through RNA silencing [2]. RNA silencing refers to a variety of mechanisms where a small RNA molecule interferes with a given nucleotide sequence. Plant RNA silencing operates via RNA-directed DNA-methylation (RdDM) to repress transcription or by targeting mRNAs via post-transcriptional gene silencing (PTGS) [3]. 

RNA silencing is triggered by double-stranded RNA (dsRNA), and the generation and function of the small RNAs depend on key protein families such as Dicer-like (DCLs), Argonautes (AGOs), and RNA-dependent RNA polymerases (RDRs) [4]. The RNA silencing pathways rely on distinct DCL proteins that cleave dsRNA precursors into small RNAs 21–26 nucleotides in length [5], small-interfering RNAs (siRNAs), or microRNAs (miRNAs) [6]. In *Arabidopsis thaliana*, dsRNAs are processed into specifically-sized sRNA duplexes by one of the four DCL (AtDCL1–4) proteins. dsRNA processing, called dicing, is facilitated by one of the six dsRNA-binding proteins (HYPONASTIC1 or AtHYL1, AtDRB2–5, and AtDRB7) that interact with specific DCLs [7,8]. dsRNA might derive directly from virus replication, inverted repeats, or convergent transcription. dsRNA formation may also be genetically programmed at endogenous loci that produce transcripts with internal stem-loop structures. Alternatively, in *A. thaliana*, dsRNA may be synthesized by one of the six RDRs (AtRDR1–6) that copy single-stranded RNA (ssRNA) to initiate a new round of RNA silencing. These small RNAs are then incorporated into AGO-containing RNA-induced silencing complexes (RISCs) that guide small RNAs to their targets by sequence complementarity resulting in target RNA degradation, translational inhibition, or heterochromatin formation [6]. The *A. thaliana* genome encodes 10 AGO proteins (AGO1-10), with various functions such as implication in the RdDM pathway (AGO4) or viral defense (AGO2). 

RdDM requires specialized transcriptional machinery centered on two plant-specific RNA polymerase II (Pol II)-related enzymes called Pol IV and Pol V [9]. Pol II, Pol IV, and Pol V have each have 12 subunits. Half of these subunits are common in Pols II, IV, and V, but each Pol also has specialized subunits. Subunits are named nuclear RNA polymerase B (NRPB) for Pol II subunits, NRPD for Pol IV subunits, and NRPE for Pol V subunits. The largest specialized subunits in Pol IV and Pol V are NRPD1 and NRPE1, respectively, and they bind to a shared subunit NRPD2/NRPE2 to form the catalytic cores [9]. NRPD1 and NRPE1 differ from NRPB1 by many substitutions or deletions of conserved amino acids, which probably contribute to their specialized functions in RdDM. Pol IV and Pol V are essential for the biogenesis and function of heterochromatic (hc)-siRNAs, which mediate TGS by RdDM (or histone modification) [10].

The availability of an increasing number of plant genomes shows that there is a large variation in the number of gene members of the core families encoding key components of RNA silencing. For example, *A. thaliana*, rice, tomato, soybean, and *Medicago truncatula* present four, eight, seven, five, and six *DCL* genes, respectively [11,12,13,14,15]. Similarly, the *AGO* gene family has expanded from three members in green algae [16] to 6 in moss, 10 in *Arabidopsis,* 17 in maize, 19 in rice, 25 in tomato, 22 in soybean, 27 in *Brassica napus,* and 11 in potato and coffee [12,13,15,17,18,19,20,21,22]. Plant AGO proteins are grouped into three major clades: AGO1/5/10, AGO2/3/7, and AGO4/6/8/9 [17,23]. These phylogenetic analyses showed that the diversification of the *AGO* gene family is an ancient and probably continuous process. This could mirror a functional diversification of AGO and DCL proteins, presumably reflecting expanding small RNA-directed regulatory pathways [17]. Likewise, the RDR family has also been expanded in different plant species, for example, from 6 members in rice and tomato to 7 and 16 in soybean and *B. napus,* respectively [12,13,15,24].

Common bean (*P. vulgaris*) is the most important grain legume for direct human consumption in the world, particularly in developing countries where it constitutes an important source of protein and essential micronutrients [25]. Unfortunately, bean production can be drastically impaired by environmental conditions and particularly by fungal diseases. Anthracnose, caused by the hemibiotrophic fungal pathogen *C. lindemuthianum*, is one of the most widespread and economically important diseases [25,26]. Common bean is an autogamous diploid (2n = 2x = 22) species with a relatively small genome (∼630 Mb) [27]. *P. vulgaris* is not only a major pulse crop, but is also an ideal model for crop evolutionary studies because of its complex evolution, which led to two major gene pools known as the Andean and Meso-American gene pools [28]. The divergence between these two gene pools was estimated to have occurred ca. 110 000 to 165 000 years ago [29,30]. In that context, two genome assemblies of the common bean are available, one for genotype G19833 of Andean origin [30] and one for genotype BAT93 of Mesoamerican origin [31]. *AGO* and *DCL* genes have been analyzed in the Andean G19833 genotype leading to the identification of 15 *PvAGO* genes and 6 *PvDCL* genes [32]. Consequently, except for the report of de Sousa Cardoso et al. [32], our knowledge of the RNA silencing mechanism in common bean remains quite poor.

The aims of this study were to identify and characterize, by in silico analysis, the genes involved in RNA silencing, including *AGO*, *DCL*, *RDR*, *DRB*, *NRPD1*, *NRPE1,* and *NRPD2/NRPE2* in common bean. Taking advantage of the availability of two genome assemblies of contrasting origins (Andean and Mesoamerican), we wanted to address the evolution of these genes on a short time scale. Their expression patterns were investigated in different organs using online data and after infection with the fungus *C. lindemuthianum* by quantitative RT-PCR. The identification of these core components to trigger RNA silencing in this non-model plant species of worldwide economic relevance pave the way for further investigation.

## 2. Materials and Methods

### 2.1. Common Bean Genome Sequence Databases and Annotation Data

G19833 (v1.0) and BAT93 (v10) *P. vulgaris* genome assemblies and annotation data were downloaded from Phytozome (v10.0) (http://www.phytozome.net/, accessed on 1 December 2020) and from BAT93 genome data repository [31] (http://denovo.cnag.cat/genomes/bean/, accessed on 1 December 2020), respectively.

### 2.2. Identification of Argonaute, Dicer-like, RDR, DRB, NRPD1, NRPE1, and NRPD2 Genes in Common Bean Genomes

In order to identify *DCL*, *AGO*, *RDR*, *DRB*, *NRPD1*, *NRPE1*, *NRPD2* genes, tBLASTn [33] search was performed on the G19833 and BAT93 genome sequences with the published Arabidopsis *DCL* [34], *AGO* [17], *RDR* [15], *DRB* [15], *NRPD1*, *NRPE1,* and *NRPD2* [35] gene sequences as queries, using a cut-off E-value of 1e-10. Gene structure was determined by integrating evidence in the Artemis annotation platform [36], including (1) Genemark.hmm *ab initio* gene prediction [37], (2) *Glycine max* and *P. vulgaris* ESTs available from Genbank, aligned on the genomes using Sim4 [38], (3) similarities to protein sequences identified using BLASTx [33] on *G. max* (Wm82.a2v1) from Phytozome (v10.0) and *Arabidopsis* (TAIRv10) (https://www.arabidopsis.org, accessed on 1 December 2020), (4) contigs resulting from G19833 RNA-seq velvet assembly [30,39] aligned on both G19833 and BAT93 genomes using Sim4 [38]. Finally, the Pfam database (http://pfam.xfam.org/, accessed on 1 December 2020) was used to confirm each candidate gene by checking the presence of the typical domain of each family. DCL proteins should have an N-terminal helicase domain (DExD/H-box and helicase-C subdomains) followed by DUF283 (domain of unknown function, known also as Dicer dimerization domain), PAZ (Piwi-Argonaute-Zwille), tandem RNase III domains, and one or two C-terminal double-stranded RNA binding domains (dsRBDs) [14]. AGOs should have PAZ, MID (middle), and PIWI domains. RDRs should have a conserved RDRP domain. DRB proteins should have two double-stranded RNA binding motif domains. 

Candidate proteins were named based on their phylogenetic proximity to known members in *A. thaliana*, soybean, and/or *M. truncatula*. The prefix PvA or PvM was added for sequences originating from G19833 (Andean) or BAT93 (Meso-American), respectively.

### 2.3. Protein Sequence Alignment and Phylogenetic Tree Building

The complete sequence of each putative AGO, DCL, RDR, and DRB proteins were aligned using Muscle [40], and the resulting alignments were manually optimized using SeaView [41]. For a given gene, when more than one isoform was identified, the longest was selected for the alignment. Aligned sequences were then analyzed using ProtTest3 [42] to estimate the best phylogenetic model. Maximum-likelihood trees were generated with PhyML [43]. Bootstrap values were computed with the consensus of 1000 trees generated with PhyML. The resulting phylogenetic trees were displayed using MEGA version 7 [44]. For phylogenetic analysis, the common bean sequences were completed with AGO sequences from soybean [17], DCL sequences from soybean, and *M. truncatula* [14], and RDR and DRB1 [also known as HYPONASTIC LEAVES 1 (HYL1)] sequences from soybean [15].

### 2.4. Characterization of the P. vulgaris DCL, AGO, RDR, DRB, NRPD, and NRPE Genes

The location of each *PvA_AGO, PvA_DCL, PvA_RDR, PvA_DRB, PvA_NRPD, PvA_NRPE* gene on G19833 chromosomes was determined by tBLASTn searching against the G19833 genome. Molecular weights (Mol. Wt.) and isoelectric points (pI) were determined using the Pepstats program from EMBOSS [45] analysis package. The number of isoforms in G19833 (v1.0) and BAT93 (v10) were obtained from corresponding official annotations in the Phytozome (V9.0) and BAT93 genome data repositories, respectively. Protein similarity and identity percentage were calculated with needleglobal pairwise alignment [45]. The number of introns in the CDS was obtained from manual reannotation performed in the Artemis platform [35].

### 2.5. RNA-Seq Data Analysis

RNA-seq data from G19833 genotype, were downloaded at https://www.ncbi.nlm.nih.gov/sra?linkname=bioproject_sra_all&from_uid=41439 (accessed on 1 December 2020), for 11 different organs including: roots_10DAP (days after planting), trifoliates_19DAP, young_pods, Leaves_10DAP, stem_10DAP, stem_19DAP, nodules_19DAP, roots_19DAP, mature_pods, flower_buds, flowers [30]. RNA-seq count data were transformed as moderated log-counts-per-million using the package EdgeR (version 3.16.4, [46]) in the statistical software ‘R’ (version 3.3.2, [47]). Then for each subset of genes, we used the MixOmics R package (version 6.1.1, [48]) to run a hierarchical clustering on both genes and organs using the Euclidean distance and Ward method. 

### 2.6. Plant Materials, Infection with C. lindemuthianum, RNA Extraction, and RT-qPCR Analysis

Infections of the common bean Andean landrace JaloEEP558 with the incompatible strain 100 of *C. lindemuthianum* were carried out as previously described in Richard et al. [49]. A time-course gene expression analysis was conducted at 6, 24, 48, 72, and 96 hpi in JaloEEP558 seedlings infected with strain 100. For each time, one of the two cotyledonary leaves from three different inoculated plants and control plants were sampled and flash-frozen in liquid nitrogen for RNA isolation and RT-qPCR analysis.

Total RNA extraction and quantitative RT-PCR (RT-qPCR) experiments were performed as described in Richard et al. [49]. The expression analyses of the genes *PvAGO1*, *PvAGO2a*, *PvDCL2a*, *PvDCL2b*, *PvAGO4a*, *PvAGO4b,* and *PvAGO4c* were performed using the gene-specific primers listed in Supplementary Table S1. Gene expression was normalized with four reference genes (*PvUkn1*, *PvUkn2*, *PvIDE,* and *PvAct11*) [50] (Supplementary Table S1). For each gene, gene expression in mock condition was used to calibrate gene expression in infected plants at each time point. Relative gene expression in inoculated leaves compared to mock leaves was calculated using the method 2^−ΔΔCt^ on three technical replicates and two biological replicates [51]. Statistical comparisons were carried out using unpaired *t*-tests between each mean value (at *t* = 6, 24, 48, 72, 96 hpi) and the corresponding mean value at *t* = 0 hpi.

## 3. Results

### 3.1. Six Putative DCL Genes Are Present in P. vulgaris Genome

**Figure 1:**
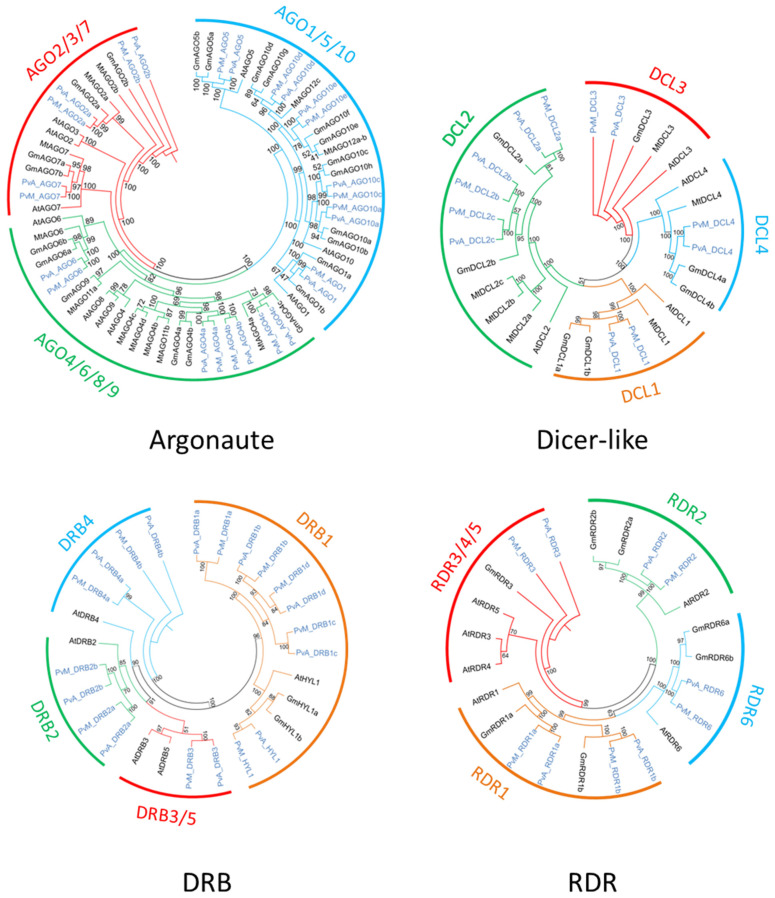
Phylogenetic analysis of Argonaute, Dicer-like, DRB, and RDR family. *Pv* sequences are presented in light blue, while sequences from *A. thaliana*, soybean, and *M. truncatula* are presented in black.

**Figure 2:**
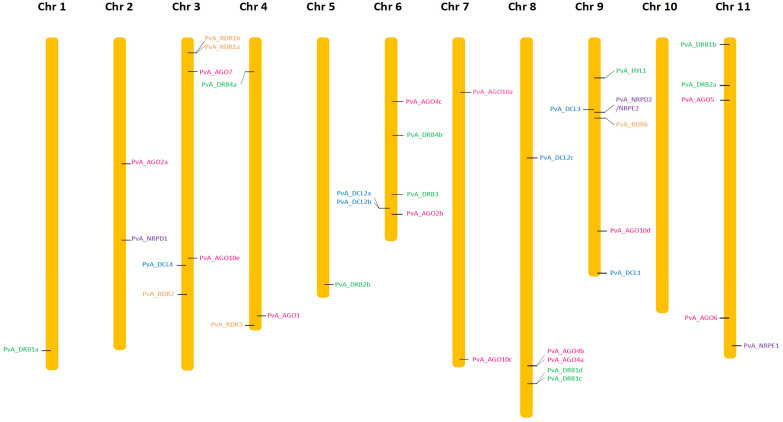
Chromosomal localization of *AGO* (pink), *DCL* (light blue), *DRB* (green), *RDR* (orange), *NRDP1*, *NRPE1*, and *NRPD2*/*NRPE2* (purple) genes in the common bean genome (G19833).

The search for homologous DCL sequences in the *P. vulgaris* genome generated six full-length *DCL* genes recovered from both G19833 and BAT93 genomes (Table 1). These genes were named using the prefix PvA_ or PvM_ to indicate genotype G19833 (Andean) or BAT93 (Meso-American), respectively, or PvA/M to indicate a gene present in both genotypes. PvA/M prefix was then followed by an identifier for their *Arabidopsis* homologs determined by phylogenetic analysis (e.g., *PvA_DCL1* corresponds to the *AtDCL1* gene). For paralogs, a letter (a, b, c…) was used as the suffix. The same nomenclature was used for all genes involved in RNA silencing described in this study. Dicer-like 1–4 occurred as monophyletic groups containing DCLs from *P. vulgaris*, *G. max*, *M. truncatula,* and *A. thaliana*. Our manual annotation allowed us to identify *PvM_DCL2c* that was not present in the automatic annotation of BAT93 assembly. In *P. vulgaris*, for both BAT93 and G19833, *DCL1*, *DCL3*, and *DCL4* occurred as single-copy genes, while DCL2 had three paralogs (*PvA/M_DCL2a*, *PvA/M_DCL2b*, *PvA/M_DCL2c*) (Figure 1, Table 1). The six *DCL* genes in the common bean presented high levels of protein identity between BAT93 and G19833 (> 97% protein identity). *PvA_DCL2a* and *PvA_DCL2b* were separated by 2.5 kb on chromosome 6, while *PvA_DCL2c* was located on chromosome 8 (Figure 2). Despite their tight physical linkage, *DCL2a* and *DCL2b* were phylogenetically separated (Figure 1), such that PvA/M_DCL2b and PvA/M_DCL2c grouped with GmDCL2b, while PvA/M_DCL2a grouped with GmDCL2a (Figure 1). Manual inspection of flanking genes in the *P. vulgaris* and *G. max* genomes showed that both copies of *DCL2* (*a* and *b*) are located in a syntenic region (Supplementary Figure S1). Indeed, in both species, the duplicated *DCL2* genes were flanked by genes encoding a histidinol dehydrogenase and a protein male sterile 5 on one side and by genes encoding a stress up-regulated Nod 19 and 3-hydroxyisobutyrate dehydrogenase on the other side (Supplementary Figure S1). Amplification of *DCL2* genes has also been observed in *M. truncatula,* which has three copies [14]; however, these DCL2s formed a separate clade (Figure 1). The PvA/M_DCL proteins ranged in length from 1388 to 1975 amino acids (aa) (Table 1). As observed for other legume species, the smaller DCL proteins occur within the DCL2 clade [52].

### 3.2. Thirteen AGO Genes in Common Bean Genome

The search for homologous AGO sequences in the *P. vulgaris* genome generated 13 full-length *AGO* genes recovered from both G19833 and BAT93 genomes (Table 1). Our manual annotation allowed us to correct *PvM_AGO2a* by fusing two distinct genes from BAT93 automatic annotation leading to a 971 aa long PvM_AGO2a protein, sharing 99.3% of protein identity with the G19833 homolog (Table 1). The length of the identified AGOs varied from 886 to 1063 aa. The *Pv AGO* genes were spread on 8 out of 11 common bean chromosomes, with two genes (*PvA_AGO4a* and *PvA_AGO4b*) organized in a tandem array on chromosome 8 and separated by ∼20 kb (Figure 2). The phylogenetic tree classified the AGOs proteins into three clades: AGO 1/5/10, AGO 4/6/8/9, and AGO 2/3/7 (Figure 1). For each 13 *Pv AGO* genes, a clear orthology relationship was identified between G19833 (*PvA_AGO*) and BAT93 (*PvM_AGO*), testifying to the absence of recent gene duplication or deletion for this AGO gene family (Figure 1). In particular, the gene duplication leading to *PvA/M_AGO4a* and *PvA/M_AGO4b* occurred prior to the Andean/Mesoamerican gene pool divergence.

### 3.3. Five RDR Genes in the Common Bean Genome

Common bean G19833 and BAT93 genomes contain five *RDR* genes each (Table 1), located on chromosomes 3, 4, and 9 (Figure 2). Our manual annotation allowed us to correct *PvM_RDR1a* by fusing two distinct genes from BAT93 automatic annotation leading to an 1139 aa long PvM_RDR1a protein, sharing 99.3% of protein identity with its G19833 homolog (Table 1). The length of RDRs ranged from 980 aa to 1222 aa (Table 1). As previously observed [12,53], phylogenetic analysis grouped RDR into four clades (RDR1, RDR2, RDR3, RDR6) with clade RDR3 containing three *Arabidopsis* members (AtRDR3, AtRDR4, AtRDR5) out of the 6 AtRDR (Figure 1). Concerning *P. vulgaris*, each clade contained a single *Pv RDR* gene, except for clade 1, which contained two *RDR1* paralogs (*PvA/M_RDR1a* and *PvA/M_RDR1b*) closely linked on chromosome 3 and separated by 10 kb (Figure 1 and Figure 2). Similarly, two *RDR1* paralogs were also identified in chickpea and pigeonpea genomes [50], suggesting that they could correspond to an ancient gene duplication.

### 3.4. Ten DRB Genes in Common Bean Genome

Ten *DRB* genes were identified in both G19833 and BAT93 genomes (Table 1) with a clear orthology relationship, suggesting no recent duplication/deletion for this gene family in common bean (Figure 1). Our manual annotation led us to correct *PvM_DRB4b* by fusing two distinct genes from BAT93 automatic annotation leading to a 248 aa long PvM_DRB4b protein, sharing 99.6% of protein identity with its G19833 homolog (Table 1). Compared to *Arabidopsis*, the common bean genome experienced an amplification of the *DRB1* gene family (five members) as well as the *DRB4* gene family (two members). A clear ortholog of *AtHYL1*, a key interactor of DCL1 in miRNA biogenesis [54], referred to as *PvA/M_HYL1*, was identified on common bean chromosome 9 (Figure 1 and Figure 2). The 10 common bean *DRB* genes were spread on seven chromosomes, with *PvA/M_DRB1d* and *PvA/M_RDB1c* genes tightly linked on chromosome 8.

### 3.5. Common Bean Pol IV and Pol V

In order to gain insight into the Pol IV and Pol V complex in the common bean genome, the largest and second-largest subunits of Pol IV and Pol V were searched by seeking AtNRPD1, AtNRPE1, and AtNRPD2/NRPE2 against common bean BAT93 and G19833 genomes with tBLASTn. Common bean encodes one NRPD1, one NRPE1, and one NRPD2/NRPE2, and hence they are named *PvA/M_NRPD1*, *PvA/M_NRPE1,* and *PvA/M_NRPD2/NRPE2* (Table 1). These three proteins present a high level of identity (> 99%) between BAT93 and G19833. They are located on chromosome 2 (*PvA_NRPD1*), 11 (*PvA_NRPE1*), and 9 (*PvA_NRPD2/NRPE2*) (Figure 2).

### 3.6. In Silico Expression Pattern of AGO, DCL, RDR, DRB, NRPD1, NRPE1, and NRPD2 Candidate Genes

**Figure 3:**
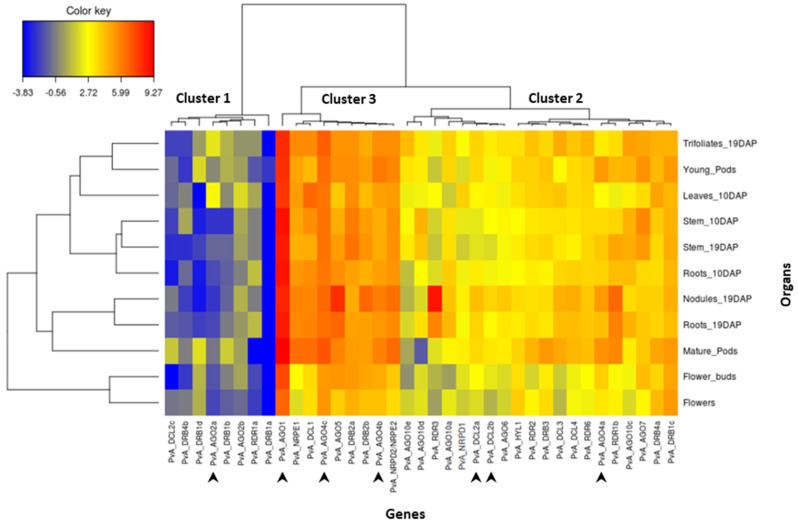
Heat map showing the expression pattern of *PvA_AGO, PvA_DCL, PvA_RDR, PvA_DRB, PvA_NRDP1, PvA_NRPE1, and PvA_NRPD2/NRPE2* genes in 11 common bean organs from genotype G19833. The color scale fold-change values are shown on the left of the heat map. DAP = days after planting. Arrows indicate genes analyzed in RT-qPCR experiments after *C. lindemuthianum* infection.

In order to analyze the transcript abundance of these 37 genes in different organs of common bean, we performed a comprehensive gene expression in silico analysis using online RNAseq data for genotype G19833. The results are shown in Figure 3. After moderated log-counts-per-million transformation, we applied hierarchical clustering (with Euclidean distance and Ward method) on the 37 genes. The genes can be organized into three clusters. Cluster 1 corresponds to genes presenting a low expression level. This cluster comprises several *DRB* genes (*PvA_DRB4b*, *1d*, *1b*, *1a*), two AGO genes (*PvA_AGO2a*, *2b*), one *DCL* gene (*PvA_DCL2c*), and one *RDR* gene (*PvA_RDR1a*). Cluster 3 corresponds to genes that are highly expressed and comprises four *AGO* genes (*PvA_AGO1*, *4c*, *5*, *4b*), two *DRB* genes (*PvA_DRB2a*, *2b*), one *DCL* gene (*PvA_DCL1*), as well as *PvA_NRPE1* and *PvA_NRPD2*. In particular, *PvA_AGO1* seems to be highly expressed in all tested organs. Finally, the remaining 20 genes correspond to genes presenting an intermediary expression level (cluster 2; Figure 3). For most genes of this cluster, the expression level seems relatively homogenous in the 11 analyzed organs, except *PvA_RDR3,* which seems up-regulated in the nodules.

### 3.7. Expression Pattern Analysis after Fungus Infection

**Figure 4:**
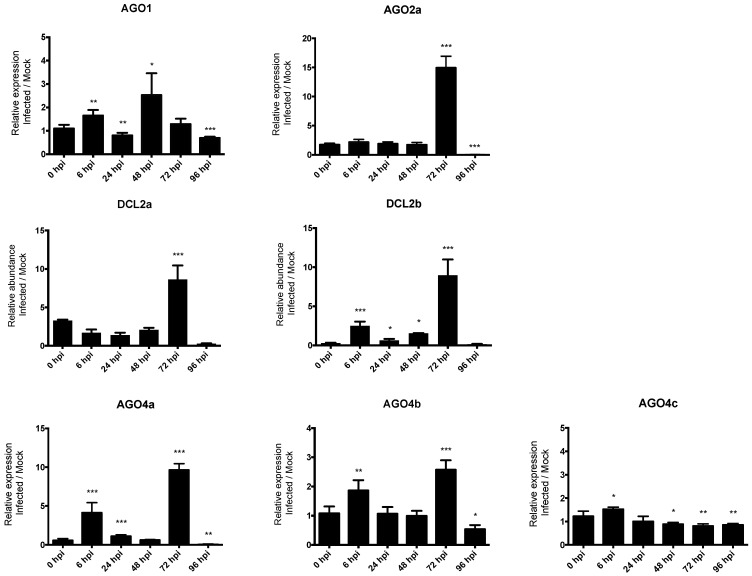
The expression levels of *P. vulgaris* AGO and DCL genes in response to *C. lindemuthianum* infection. Bars show means +/− SD. * *p* < 0.05; ** *p* < 0.01; *** *p* < 0.001, unpaired *t*-test.

In order to investigate the role of RNA silencing in pathogen defense in common bean, we studied the expression profile of seven genes, including *AGO1, AGO2a, DCL2a, DCL2b, AGO4a, AGO4b*, and *AGO4c* (indicated by the arrows in Figure 3). The expression of these genes in response to infection with the hemibiotrophic fungus *C. lindemuthianum* was studied using RT-qPCR at 6, 24, 48, 72, 96 hpi in a resistant genotype (incompatible interaction). Significantly, temporal gene expression analysis revealed that *DCL2a* and *DCL2b* are both ca. nine-fold up-regulated after infection compared to the mock control at 72 hpi. Similarly, a clear upregulation is observed at 72 hpi for both *AGO4a* and *AGO2a*, and also for *AGO4b* but with a lower extend (Figure 4). Conversely, the expression of *AGO1* and *AGO4c* was not modified upon *C. lindemuthianum* infection (Figure 4).

## 4. Discussion

Several studies have pointed out that genes involved in silencing evolve rapidly with a great variation of number even between closely related species. However, our comprehensive analysis of various gene families involved in RNA silencing in two common bean genomes of contrasting origins allowed us to identify the same number of *AGO* (13), *DCL* (6), *DRB* (10), *RDR* (5), *NRPD1* (1), *NRPE1* (1), and *NRPD2/NRPE2* (1) genes in both G19833 (Andean) and BAT93 (Meso-American) genomes, suggesting that no recent gene duplication/deletion occurred after gene pool divergence. Indeed, for each of the 37 genes analyzed in the present study, orthologs presenting a high percentage of protein identity (> 94%) were unambiguously identified between BAT93 and G19833 (Table 1, Figure 1). This suggests that even if the genome assembly of BAT93 is of lower quality compared to G19833, and does not allow repeated sequence analysis [55], this quality is sufficient for gene analysis. However, we can not exclude that some genes were missing, and higher quality genome assembly based on long-read sequencing will be needed to unambiguously address this question. These 37 genes are distributed on all *Pv* chromosomes, except chromosome 10. Importantly, our manual annotation led us to correct misannotated genes, in particular in BAT93 (Table 1). Even if no recent gene dynamics were identified after gene pool divergence, an interesting pattern of evolution was identified for DCL and AGO gene families in the common bean. 

*DCL* genes, and in particular *DCL2* genes, present a complex pattern of evolution in legume species. Unlike the single-copy genes of *DCL1*, *DCL3,* and *DCL4*, in *Pv* and *Mt,* there were three copies of *DCL2*. Soybean contains seven *DCL* genes in its ancient polyploid (paleopolyploid) genome [56] (Figure 1). In soybean, the most recent genome doubling event occurred approximately 9–14 million years ago, and the soybean genome maintains at least one gene duplicate for ca. 75% of its genes, termed homoeologous gene pairs [57]. *GmDCL4a*/*GmDCL4b* and *GmDCL1a*/*GmDCL1b* correspond to such homoeologous gene pairs, while *GmDCL3* is present as a single copy. By contrast, *GmDCL2a* and *GmDCL2b* are locally duplicated, separated by 5 kb, on chromosome 9. In soybean, the age of this *GmDCL2a/GmDCL2b* duplication was estimated to be 19.4 Mya [56], indicating that it predates the whole genome duplication of soybean at 9–14 Mya [56], and the split for common bean and soybean 19 Mya [58,59]. Consequently, this strongly suggests that in the putative *Pv Gm* common ancestor, a locally duplicated pair of *DCL2* genes were present. In agreement with this, we found in common bean two *DCL2* genes, *Pv_DCL2a* and *Pv_DCL2b,* organized in tandem array in the corresponding syntenic region with soybean (Figure 2 and Figure S1). In the *Pv* genome, an additional paralog, *DCL2c*, present on chromosome 8, was putatively derived from *Pv_DCL2b* by a yet unknown mechanism that could involve transposable elements identified in the vicinity of *DCL2c*. Three copies of *DCL2* were also identified in *Mt* [14]. However, this amplification appears to be independent of that observed in the common bean (Figure 1). In contrast, only a single *DCL2* has been identified in various other legume species, including chickpea (*Cicer arietinum*), pigeonpea (*Cajanus cajan*), and each of the two genomes composing the allotetraploid groundnut (*Arachis duranensis* and *Arachis ipaensis*) [52]. These independent amplifications of the *DCL2* genes in specific legume species could lead to their functional diversification and probably reflect their functional importance. In *Mt*, a nodule-specific role for DCL2 has been proposed [14], while in soybean, *DCL2* genes regulate traits such as seed color *via* the production of 22 nucleotide siRNA from long inverted repeats [60]. In another study, *GmDCL2* paralogs exhibited a wide range of transcriptional changes in response to stress, suggesting *DCL2*s may play an important role in stress response [56]. Congruent with these findings, we found that *Pv_DCL2a* and *Pv_DCL2b*, mildly expressed in most organs (Figure 3), are up-regulated at 72 hpi in leaves infected by *C. lindemuthianum* (Figure 4).

We identified 13 AGO genes in *Pv*, while 15 AGO genes were reported in a previous analysis performed on the G19833 genome. Indeed, compared to de Sousa Cardoso et al. [32], our manual annotation led us to discard one AGO10 and one AGO2 gene. Similarly, 13 AGO genes were also identified in both chickpea and pigeonpea [52]. Within angiosperms, several AGO subgroups have expanded differently in monocots and eudicots, with lineage-specific gene duplications [61]. For example, the grasses exhibit an expanded AGO 1/5/10 clade [17]. More precisely, maize and rice harbor many AGO5 paralogs, and a grass-specific AGO18 family, a deep branch of the AGO1/5/10 clade, has been discovered and played important roles during plant reproduction and viral defense [17]. In common bean, expansion of the AGO1/5/10 clade was also observed, but it was the result of AGO10 gene amplification since four AGO10 genes were identified in the common bean genome (Table 1). Similar amplification of AGO10 was also observed in soybean, where eight paralogs were identified in its paleopolyploid genome [17]. Each *Pv* AGO10 gene clearly corresponds to two *Gm* orthologs (Figure 1), strongly suggesting that AGO10 amplification occurred prior to the soybean/common bean divergence. In soybean, the expansion of the AGO10 family presumably co-evolved with the expansion of the miR165/166 family since 21 copies of miR165/166 are annotated in the soybean genome [17]. Likewise, expansions of miR165/166 genes, with 10 copies, have also been identified in the *Pv* genome (Geffroy V. and Meyers B.C.; unpublished results). In addition to AGO10, expansion was also observed for AGO2 (two members) and AGO4 (three members) in the *Pv* genome. *At*AGO4 primarily binds 24-nt, repeat and heterochromatin-associated siRNAs, and functions in RNA-directed DNA methylation [62], while *At*AGO2 functions in antibacterial immunity [63]. In common bean, AGO2 and AGO4 genes have non-redundant expression profiles (Figure 3), suggesting that they may have acquired divergent functions. *PvA_AGO1* seems to be highly expressed in all common bean-tested organs. In agreement with our results, AGO1 expression is detected in many organs, such as leaves, roots, and flowers, in *Arabidopsis* [64], rice [12], *B. napus* [65], and the emerging medicinal model plant *Salvia miltiorrhiza* [66].

Functional analysis of genes involved in RNA silencing revealed that most of them play multiple roles, not only in growth and development but also in immune defense against pathogens [1,67,68,69]. The importance of RNA silencing in plant viral defense has been well documented for a long time [63]. In addition to viral defense, more and more evidence is accumulating, showing that RNA silencing also plays a role in plant interactions with bacterial pathogens [70]. More recently, the potential role of RNA silencing in plant defense has also been reported for several fungal pathogens, including *Verticillium dahliae* [71], *Verticillium longisporum* [72], *Magnaporthe oryzae* [73], and *Botrytis cinerea* [74]. The importance of RNA silencing in plant defense is illustrated by the fact that it has stimulated a counter-defense system from the pathogens to overcome it. Indeed, it is now well-known that pathogens of a different nature (viruses, bacteria, fungi, oomycetes, and phytoplasma) have evolved effectors that are able to target and suppress the host plant RNA silencing pathway [67,75,76,77,78]. Suppressors of RNA silencing were first discovered in viruses (VSRs, for viral suppressors of RNA silencing) [4]. At present, there is no evidence of putative suppressors of silencing acting in *C. lindemuthianum*. However, there is growing evidence that this is a common mechanism exploited by fungal pathogens to promote their infection [79]. Consequently, such suppressors probably exist in *C. lindemuthianum,* although not yet identified.

To investigate the contribution of some of the genes involved in RNA silencing in the defense response in common bean, we performed quantitative RT-PCR-based expression analysis on leaves of resistant bean plants inoculated with the hemibiotrophic fungus *C. lindemuthianum* (in an incompatible context). Whereas expression levels of *PvA_AGO2a*, *PvA_AGO4a,* and *PvA_DCL2* (*a* and *b)* is low to moderate without any biotic stress (Figure 3), a strong up-regulation of these genes was observed mainly at 72 hpi (Figure 4). On the contrary, expression of *PvA_AGO1* and *PvA_AGO4c*, which are ubiquitously and highly expressed in uninfected plants, was not significantly modified after infection. This suggests that after fungal infection, *PvA_AGO2*, *PvA_AGO4,* and *Pv_DCL2* may play a prominent role in the sRNA-based regulation of defense gene expression in the common bean. Interestingly, the Argonaute proteins, AGO4 and AGO2, have both been linked to antibacterial defense. AGO4, a component of the RdDM pathway that directs DNA methylation at specific loci, mediates resistance to *P. syringae*, independently of the other components of the RdDM pathway [80]. AGO2 functions in antibacterial immunity by binding a specific miRNA to modulate the exocytosis of antimicrobial PR proteins [81]. In the literature, different pathosystems involving either hemibiotrophic pathogens or incompatible plant-microbe interactions present similar results. Notably, in susceptible wild tobacco plants challenged by the hemibiotrophic fungus *Fusarium brachygibbosum* as well as in resistant cowpea plants in response to CPSMV (*Cowpea severe mosaic virus*) infection, an increased expression of *AGO4* has been reported, whereas no change in expression was observed for *AGO1* [82,83]. Moreover, an up-regulation of *AGO2* expression after infection was reported in *Arabidopsis* after infection by the biotrophic bacteria *P. syringae*, in the oil crop *B. napus* infected by the fungal necrotrophic *Sclerotinia sclerotiorum*, and in the cowpea *Vigna unguiculata* infected by CPSMV [20,82,84]. Concerning the role of Dicer proteins in plant defense, little is known about DCL2, except that it is involved in the processing of viral dsRNA. However, it has also been observed that the quantity of *DCL2* transcripts increases at the local site of infection by the CLRDV (*Cotton leafroll dwarf virus*) in a resistant genotype cotton *Gossypium hirsutum* [85]. This is in agreement with our results, where up-regulation of both *PvA_DCL2a* and *Pv_DCL2b* was observed in incompatible interaction with *C. lindemuthianum*. This suggests that in the common bean, an increased expression of specific genes involved in RNA silencing, acting in both the miRNA and the siRNA pathways, could counteract the infectious process of *C. lindemuthianum*. However, how these genes could regulate resistance in the common bean requires further investigation. 

## 5. Conclusions

This work will further provide a solid foundation for future functional analysis of *AGO*, *DCL, RDR, DRB, NRPD1, NRPE1*, and *NRPD2* genes in the common bean genome. For example, taking advantage of the work presented here, we would like to utilize virus-induced gene silencing (VIGS) to silence NRPD1 and NRPE1, in order to gain insight into the mechanisms involved in the unusual methylation pattern observed for NLR genes in the common bean [86,87,88]. Furthermore, our work will also help to design specific primers for RT-qPCR experiments. Finally, given the genomic location of the 37 genes studied (Table 1, Figure 2), and considering that RNA silencing is involved in a large number of traits, our work may also provide candidate genes for QTL analysis.

## Figures and Tables

**Table 1:** Identification of *AGO*, *DCL*, *RDR,* and *DRB* genes in common bean.

				Genomic Location				Protein				
Genotype	Gene Name		Accession Number	Genomic Sequence	Coordinates (5′–3′)	No. of Isoforms	ORF Length (bp)	Length (a.a.)	Mol. Wt. (Da)	pI	No. of Introns	Protein Identity ^1^
	**ARGONAUTE**											
G19833	1	*PvA_AGO1*	Phvul.004G142900	Chr04	42225570-42218739	1	3189	1063	117406.63	9.63	20	99.6%
BAT93		*PvM_AGO1*	PHASIBEAM10F025775	scaffold00773	120996-126407	1	3180	1060	117235.47	9.63	20	
G19833	2	*PvA_AGO2a*	Phvul.002G100100	Chr02	19669530-19665509	1	2913	971	109754.56	9.13	2	99.3%
BAT93		*PvM_AGO2a*	PHASIBEAM10F005923 (i)	scaffold00040	416794-413003	1	2913	971	109798.66	9.23	2	
G19833	3	*PvA_AGO2b*	Phvul.006G131700	Chr06	24674782-24679768	2	2937	979	111161.89	9.29	3	94.4%
BAT93		*PvM_AGO2b*	PHASIBEAM10F015105	scaffold00188	213085-218085	1	3078	1026	116318.64	9.02	2	
G19833	4	*PvA_AGO4a*	Phvul.008G206600	Chr08	51807602-51801232	2	2721	907	101348.73	9.25	21	100.0%
BAT93		*PvM_AGO4a*	PHASIBEAM10F016114	scaffold00210	378863-372777	4	2721	907	101348.73	9.25	21	
G19833	5	*PvA_AGO4b*	Phvul.008G206500	Chr08	51784616-51777872	1	2712	904	101388.78	9.34	21	99.8%
BAT93		*PvM_AGO4b*	PHASIBEAM10F016113	scaffold00210	357106-351920	2	2712	904	101411.82	9.33	21	
G19833	6	*PvA_AGO4c*	Phvul.006G021200	Chr06	9954415-9945414	1	2757	919	103112.92	8.86	21	99.7%
BAT93		*PvM_AGO4c*	PHASIBEAM10F001721	scaffold00005	1264498-1273271	1	2751	917	102956.73	8.76	21	
G19833	7	*PvA_AGO5*	Phvul.011G088200	Chr11	8581826-8587717	1	2985	995	109725.97	9.98	21	99.2%
BAT93		*PvM_AGO5*	PHASIBEAM10F024917	scaffold00660	91795-87085	3	2985	995	109784.06	10.04	21	
G19833	8	*PvA_AGO6*	Phvul.011G169400	Chr11	44005938-44015983	1	2658	886	99248.90	8.62	22	98.7%
BAT93		*PvM_AGO6*	PHASIBEAM10F024901	scaffold00658	56539-50355	13	2673	891	99813.45	8.52	21	
G19833	9	*PvA_AGO7*	Phvul.003G046700	Chr03	5546668-5538450	1	3081	1027	117583.48	9.50	2	99.7%
BAT93		*PvM_AGO7*	PHASIBEAM10F003253	scaffold00016	40899-38042	3	3081	1027	117553.45	9.49	2	
G19833	10	*PvA_AGO10a*	Phvul.007G062800	Chr07	5487986-5479953	1	2922	974	109675.76	9.49	20	99.9%
BAT93		*PvM_AGO10a*	PHASIBEAM10F017409	scaffold00246	117564-123835	2	2925	975	109789.86	9.48	20	
G19833	11	*PvA_AGO10c*	Phvul.007G278600	Chr07	51544600-51535965	2	2916	972	108969.37	9.53	20	96.4%
BAT93		*PvM_AGO10c*	PHASIBEAM10F022770	scaffold00482	187925-194134	9	3021	1007	112926.20	9.51	20	
G19833	12	*PvA_AGO10d*	Phvul.009G199500	Chr09	29560158-29544840	2	2724	908	103145.43	9.25	21	100.0%
BAT93		*PvM_AGO10d*	PHASIBEAM10F004033	scaffold00022	192299-198838	10	2724	908	103145.43	9.25	21	
G19833	13	*PvA_AGO10e*	Phvul.003G160000	Chr03	36714961-36722489	1	2718	906	102692.31	9.14	21	99.3%
BAT93		*PvM_AGO10e*	PHASIBEAM10F001882	scaffold00006	1336262-1342067	5	2718	906	102721.32	9.14	21	
	**DICER-like**											
G19833	1	*PvA_DCL1*	Phvul.009G260000	Chr09	37237846-37225574	1	5850	1950	218562.86	6.59	19	98.6%
BAT93		*PvM_DCL1*	PHASIBEAM10F019489	scaffold00316	98352-86768	5	5925	1975	221709.63	6.67	19	
G19833	2	*PvA_DCL2a*	Phvul.006G127100	Chr06	24163817-24176362	3	4176	1392	157780.11	7.63	21	99.6%
BAT93		*PvM_DCL2a*	PHASIBEAM10F008102	scaffold00070	816003-828602	3	4176	1392	157865.16	7.60	21	
G19833	3	*PvA_DCL2b*	Phvul.006G127200	Chr06	24178778-24194553	2	4164	1388	157241.83	7.48	21	99.5%
BAT93		*PvM_DCL2b*	PHASIBEAM10F008102	scaffold00070	837484-846206	2	4164	1388	157354.99	7.45	21	
G19833	4	*PvA_DCL2c*	Phvul.008G129500	Chr08	19880410-19869281	1	4260	1420	160912.64	7.20	22	100.0%
BAT93		*PvM_DCL2c*	(ii)	scaffold00203	292869-281676	1	4260	1420	160912.64	7.20	22	
G19833	5	*PvA_DCL3*	Phvul.009G083800	Chr09	13249918-13268354	1	5001	1667	186982.31	6.80	24	97.7%
BAT93		*PvM_DCL3*	PHASIBEAM10F014448	scaffold00174	583077-605186	1	4896	1632	182975.08	6.78	24	
G19833	6	*PvA_DCL4*	Phvul.003G175700	Chr03	38686207-38665167	1	4890	1630	183581.80	6.49	24	99.6%
BAT93		*PvM_DCL4*	PHASIBEAM10F015080	scaffold00187	512802-532487	6	4890	1630	183697.91	6.35	24	
	**RNA-DEPENDENT RNA POLYMERASE**											
G19833	1	*PvA_RDR1a*	Phvul.003G016800	Chr03	1524476-1516886	1	3417	1139	130928.53	8.60	3	99.3%
BAT93		*PvM_RDR1a*	PHASIBEAM10F010436 (iii)	scaffold00104	112619-117880	1	3417	1139	131008.64	8.60	3	
G19833	2	*PvA_RDR1b*	Phvul.003G016600	Chr03	1507885-1501098	3	3435	1145	131180.26	7.85	4	99.5%
BAT93		*PvM_RDR1b*	PHASIBEAM10F010439	scaffold00104	127700-133488	8	3366	1122	128249.56	6.70	3	
G19833	3	*PvA_RDR2*	Phvul.003G198500	Chr03	41147897-41152535	1	3357	1119	127390.10	7.27	3	99.8%
BAT93		*PvM_RDR2*	PHASIBEAM10F019797	scaffold00326	321119-325268	2	3357	1119	127374.10	7.24	3	
G19833	4	*PvA_RDR3*	Phvul.004G176400	Chr04	45666742-45687239	1	2940	980	110673.62	7.21	17	98.7%
BAT93		*PvM_RDR3*	PHASIBEAM10F011389	scaffold00117	630085-640879	4	2940	980	110803.80	7.02	17	
G19833	5	*PvA_RDR6*	Phvul.009G093700	Chr09	14423283-14418046	1	3612	1204	137676.89	7.48	1	98.4%
BAT93		*PvM_RDR6*	PHASIBEAM10F007071	scaffold00055	358845-354781	3	3666	1222	139901.52	7.53	1	
	**DOUBLE-STRANDED RNA BINDING**											
G19833	1	*PvA_HYL1*	Phvul.009G036100	Chr09	7646996-7644350	1	1059	353	38676.47	7.20	2	98.3%
BAT93		*PvM_HYL1*	PHASIBEAM10F013012	scaffold00145	297448-295070	1	1062	354	38856.65	7.11	2	
G19833	2	*PvA_DRB1a*	Phvul.001G231400	Chr01	49248540-49250360	1	1038	346	38501.96	9.85	2	95.4%
BAT93		*PvM_DRB1a*	PHASIBEAM10F007815	scaffold00066	PvA_DRB1a.fa	1	1038	346	38494.94	9.86	4	
G19833	3	*PvA_DRB1b*	Phvul.011G009300	Chr11	700663-701791	1	450	150	17025.42	8.94	2	100.0%
BAT93		*PvM_DRB1b*	PHASIBEAM10F009292	scaffold00086	599920-600849	1	450	150	17025.42	8.94	2	
G19833	4	*PvA_DRB1c*	Phvul.008G234500	Chr08	54868936-54864992	1	1095	365	38925.76	7.82	4	99.2%
BAT93		*PvM_DRB1c*	PHASIBEAM10F021174	scaffold00390	223668-220009	1	1095	365	38952.87	7.65	4	
G19833	5	*PvA_DRB1d*	Phvul.008G234400	Chr08	54863261-54860478	1	1014	338	37265.55	10.28	3	98.8%
BAT93		*PvM_DRB1d*	PHASIBEAM10F021173	scaffold00390	218135-215388	7	1014	338	37397.66	10.21	3	
G19833	6	*PvA_DRB2a*	Phvul.011G079700	Chr11	7393885-7397506	1	1230	410	44693.48	10.17	2	99.8%
BAT93		*PvM_DRB2a*	PHASIBEAM10F002315	scaffold00008	1101206-1104281	2	1230	410	44703.51	10.17	2	
G19833	7	*PvA_DRB2b*	Phvul.005G134700	Chr05	36211870-36209282	1	1230	410	44819.48	9.74	2	100.0%
BAT93		*PvM_DRB2b*	PHASIBEAM10F007902	scaffold00067	326508-328693	1	1230	410	44819.48	9.74	2	
G19833	8	*PvA_DRB3*	Phvul.006G097600	Chr06	21510646-21507124	1	1590	530	58488.76	8.89	2	97.4%
BAT93		*PvM_DRB3*	PHASIBEAM10F020740	scaffold00369	88020-85688	2	1632	544	60223.90	8.88	2	
G19833	9	*PvA_DRB4a*	Phvul.004G051700	Chr04	6519018-6524448	1	1440	480	51755.69	7.85	5	99.6%
BAT93		*PvM_DRB4a*	PHASIBEAM10F027955	scaffold01965	8266-13628	5	1434	478	51571.50	7.73	5	
G19833	10	*PvA_DRB4b*	Phvul.006G039700	Chr06	14952143-14950582	1	744	248	27621.65	6.91	2	99.6%
BAT93		*PvM_DRB4b*	PHASIBEAM10F014779 (iiii)	scaffold00182	152298-149998	1	744	248	27649.71	6.91	2	
	**Pol IV-Pol V**											
G19833	1	*PvA_NRPD1*	Phvul.002G153700	Chr02	29492370-29482158	2	4389	1463	163593.52	7.68	14	99.2%
BAT93		*PvM_NRPD1*	PHASIBEAM10F021873	scaffold00425	234429-224218	5	4416	1472	164665.81	7.73	14	
G19833	2	*PvA_NRPE1*	Phvul.011G206900	Chr11	48665190-48649579	1	6156	2052	227180.86	6.29	16	99.7%
BAT93		*PvM_NRPE1*	PHASIBEAM10F026336	scaffold00894	80124-96951	7	6156	2052	227140.81	6.33	16	
G19833	3	*PvA_NRPD2*/*NRPE2*	Phvul.009G087100	Chr09	13616163-13610899	1	3606	1202	135705.94	8.58	6	100.0%
BAT93		*PvM_NRPD2*/*NRPE2*	PHASIBEAM10F014666	scaffold00179	417378-422642	4	3606	1202	135705.94	8.58	6	

bp, base pairs; a.a, amino acid; Da, Dalton; pI, Isoelectric Point; ^1^ G19833 vs BAT93. (i). Fusion of PHASIBEAM10F005923 and PHASIBEAM10F005924. (ii). Gene not present in BAT93 annotation. (iii). Fusion of PHASIBEAM10F010436 and PHASIBEAM10F010437. (iiii). Fusion of PHASIBEAM10F014779 and PHASIBEAM10F014778.

## Data Availability

Not applicable.

## Supplementary Materials

The following are available online at https://www.mdpi.com/article/10.3390/genes13010064/s1, Figure S1: Syntenic analysis between common bean and soybean of the genomic region containing *DCL2a* and *DCL2b* genes. Table S1: List of primer sequences used in this study.
