# Supplementary material for: Genome-Wide Identification of Key Components of RNA Silencing in Two Phaseolus vulgaris Genotypes of Contrasting Origin and Their Expression Analyses in Response to Fungal Infection
Source: Genes (Basel). 2021 Dec 27;13(1):64. doi: 10.3390/genes13010064 (PMC8774654; doi:10.3390/genes13010064)
Supplement: Supplementary file 1 [file genes-13-00064-s001.zip › Figure S1.pptx]

## Slide 1
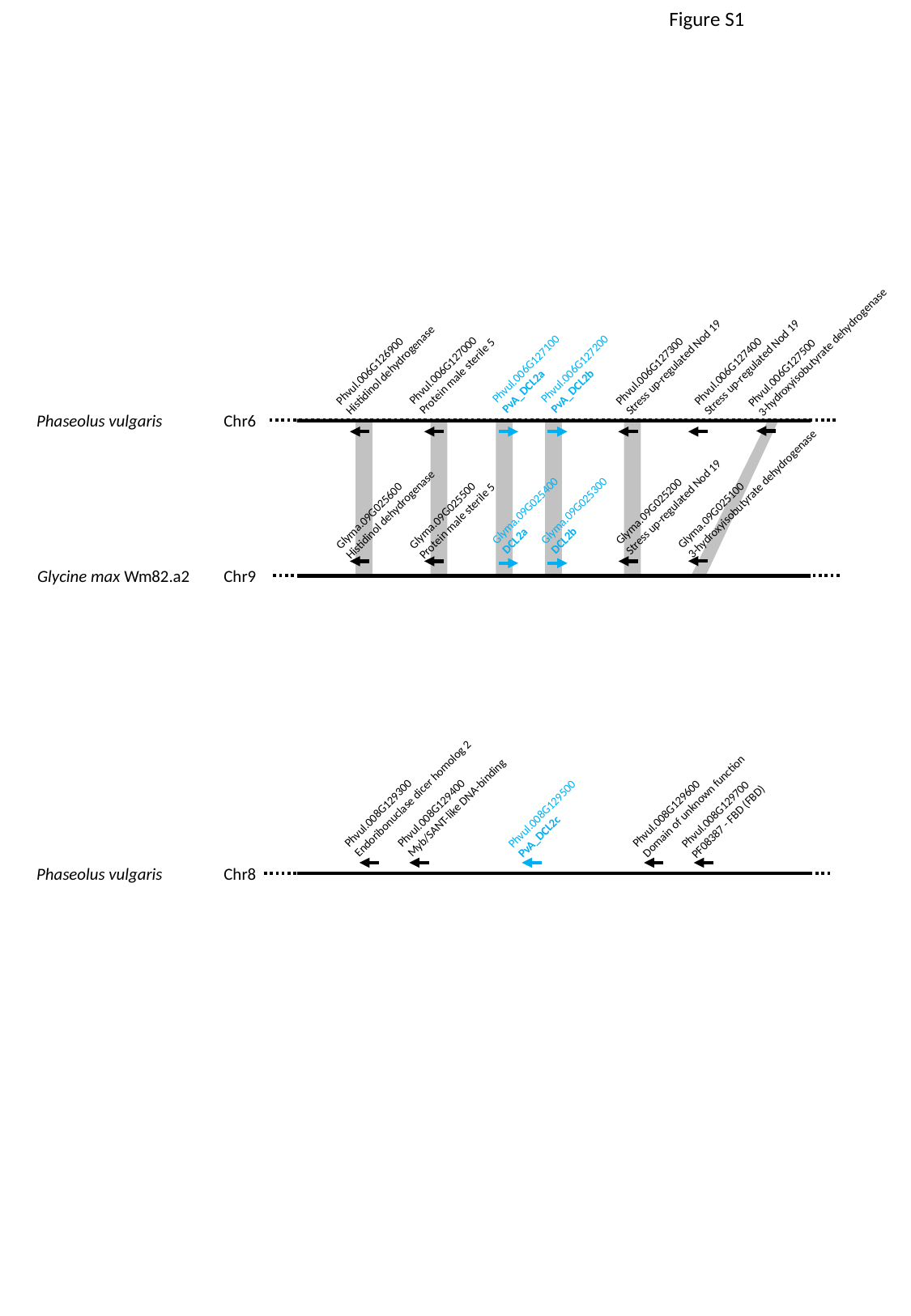

Figure S1
Phvul.006G127500
3-hydroxyisobutyrate dehydrogenase
Phvul.006G127300
Stress up-regulated Nod 19
Phvul.006G127400
Stress up-regulated Nod 19
Phvul.006G126900
Histidinol dehydrogenase
Phvul.006G127000
Protein male sterile 5
Phvul.006G127100
PvA_DCL2a
Phvul.006G127200
PvA_DCL2b
Phaseolus vulgaris
Chr6
Glyma.09G025100
3-hydroxyisobutyrate dehydrogenase
Glyma.09G025200
Stress up-regulated Nod 19
Glyma.09G025600
Histidinol dehydrogenase
Glyma.09G025400
DCL2a
Glyma.09G025300
DCL2b
Glyma.09G025500
Protein male sterile 5
Glycine max Wm82.a2
Chr9
Phvul.008G129300
Endoribonuclase dicer homolog 2
Phvul.008G129600
Domain of unknown function
Phvul.008G129400
Myb/SANT-like DNA-binding
Phvul.008G129700
PF08387 - FBD (FBD)
Phvul.008G129500
PvA_DCL2c
Phaseolus vulgaris
Chr8
