# Supplementary material for: Genome-Wide Identification of Key Components of RNA Silencing in Two Phaseolus vulgaris Genotypes of Contrasting Origin and Their Expression Analyses in Response to Fungal Infection
Source: Genes (Basel). 2021 Dec 27;13(1):64. doi: 10.3390/genes13010064 (PMC8774654; doi:10.3390/genes13010064)
Supplement: Supplementary file 1 [file genes-13-00064-s001.zip › Table S1.pdf]

**Table S1.** List of primer sequences used in this study.

| Gene name | Primer name | Primer Sequence (5′-3′) | Product length (bp) | Reference |
|---|---|---|---|---|
| PvM AGO1 | AGO1_F<br>AGO1_R | GGCAGACCTTCACCATTT<br>TGTAGTAGGGAGTTCACGGA | 106 | This study |
| PvM AGO2a | AGO2_F<br>AGO2_R | AAGATTCAGGTTACAAGTGCC<br>TGGTCCTTCGCATCATTA | 100 | This study |
| PvM AGO4a | AGO4a_2_F:<br>AGO4a_2_R: | ACCACCTGCTATCCCATCAG<br>CGCAACTTCGTCCCTTTAGA | 114 | This study |
| PvM AGO4b | AGO4b_1_F:<br>AGO4b_1_R: | TAGGGAGAAAGGTGCTCGAA<br>AGACGATTACGGGCAAGAGA | 121 | This study |
| PvM AGO4c | AGO4c 5 F<br>AGO4c_5_R | GTACGAGGACTTGTGCGTGA<br>CACCTCAAACGGCTGCTCTA | 75 | This study |
| PvM DCL2a | DCL2a_F<br>DCL2a_R | TAACACCTGAAACATTGAAGC<br>TCTTTGCGAGATACCACACT | 100 | This study |
| PvM DCL2b | DCL2b_F<br>DCL2b_R | CTGCTCTGTTGTTTATGGACT<br>CAGTTTCTCTGGTTGAATGTC | 92 | This study |
| **Reference genes** | | | | |
| Unknown 1 | Ukn1-F<br>Ukn1-R | ATTCCCATCATGCAGCAAAG<br>AGATCCCTCCAGGTCAATCC | 192 | [48] |
| Unknown 2 | Ukn2-F<br>Ukn2-R | CCAATTCAACCATCCCTCAC<br>AAACTCCTCTGCACCCTCAG | 153 | [48] |
| Actin-11 | Act11-F<br>Act11-R | TGCATACGTTGGTGATGAGG<br>AGCCTTGGGGTTAAGAGGAG | 190 | [48] |
