# Supplementary figures and images for: Genome-Wide Identification of Key Components of RNA Silencing in Two Phaseolus vulgaris Genotypes of Contrasting Origin and Their Expression Analyses in Response to Fungal Infection
Source: Genes (Basel). 2021 Dec 27;13(1):64. doi: 10.3390/genes13010064 (PMC8774654; doi:10.3390/genes13010064)

Figure S1

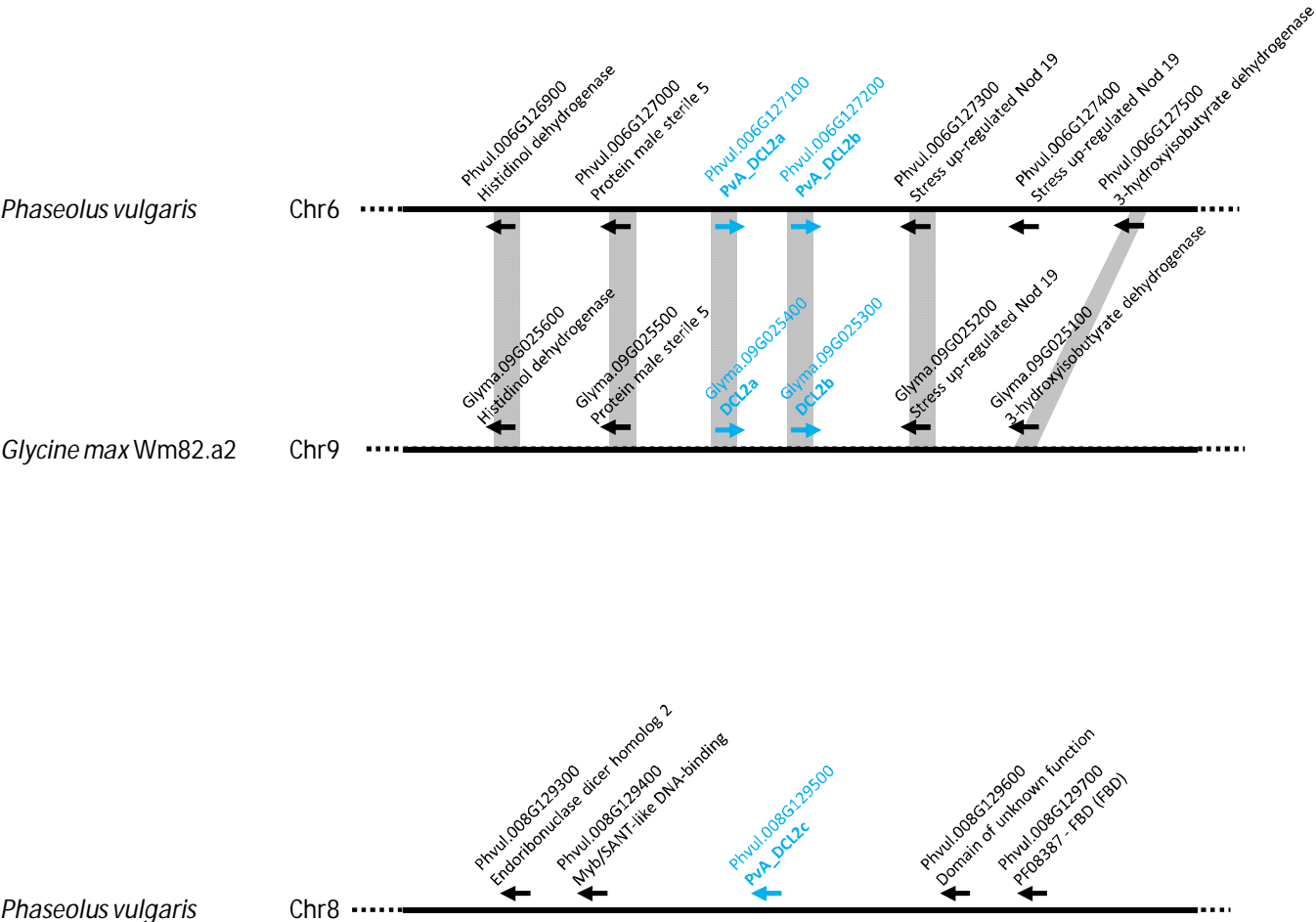

Supplement: Supplementary file 1 [file genes-13-00064-s001.zip › Figure S1.pdf]
